# Mineralogical and textural characteristics of nest building geomaterials used by three sympatric mud-nesting hirundine species

**DOI:** 10.1038/s41598-018-29307-8

**Published:** 2018-07-23

**Authors:** Dimitrios Papoulis, Olga Tzortzakaki, Pavlos Avramidis, Panagiotis Mentis, Paraskevi Lampropoulou, George Iliopoulos

**Affiliations:** 1 Department of Geology, University of Patras, Rio – Patras, 26504 Greece; 2 Department of Biology, University of Patras, Rio – Patras, 26504 Greece

## Abstract

Many hirundine species construct their nests by carrying mud particles from adjacent areas. This study aimed to investigate for the first time the materials that mud-nesting hirundines choose for nest construction from a mineralogical and sedimentological perspective. For this purpose, we sampled nests of three sympatric species, namely the Barn Swallow (*Hirundo rustica*), the Red-rumped Swallow (*Cecropis daurica*) and the House Martin (*Delichon urbicum*), from southeastern Europe. Our results showed that all species tend to use clay minerals as a cement and especially smectite and illite and if these minerals are not present in the adjacent area, they use halloysite, kaolinite or chlorite. The amounts of clay minerals in the nests are generally low indicating that the studied species can accurately identify the properties of the nesting materials. Most of the non clay minerals that they use are the common, easily accessible colourless or white minerals with low specific gravity values such as quartz, feldspars and calcite. Grain size distribution analysis revealed that the amount of clay sized grains in the mud nests of all three species is relatively low, while the amount of larger grain particles decreases when the size of the non clay minerals is small. The Red-rumped Swallow showed an increasing preference for larger grain size particles and quartz, the Barn Swallow for finer grain size particles and calcite, and the preferences of the House Martin are in between the other two species. The three hirundine species present different nest building strategies and depending on the nest architecture, each of them seems to show preference for specific minerals and specific grain sizes.

## Introduction

Swallows and martins (Hirundinidae) are small, aerial insectivorous passerines that nest either in excavated holes in banks or in cups built mainly of mud and organic material^1^. Five migratory members of this family breed in Southern Europe, i.e. the Sand Martin (*Riparia riparia*), the Crag Martin (*Ptyonoprogne rupestris*), the House Martin (*Delichon urbicum*), the Barn Swallow (*Hirundo rustica*) and the Red-rumped Swallow (*Cecropis daurica*).

**Figure 1:**
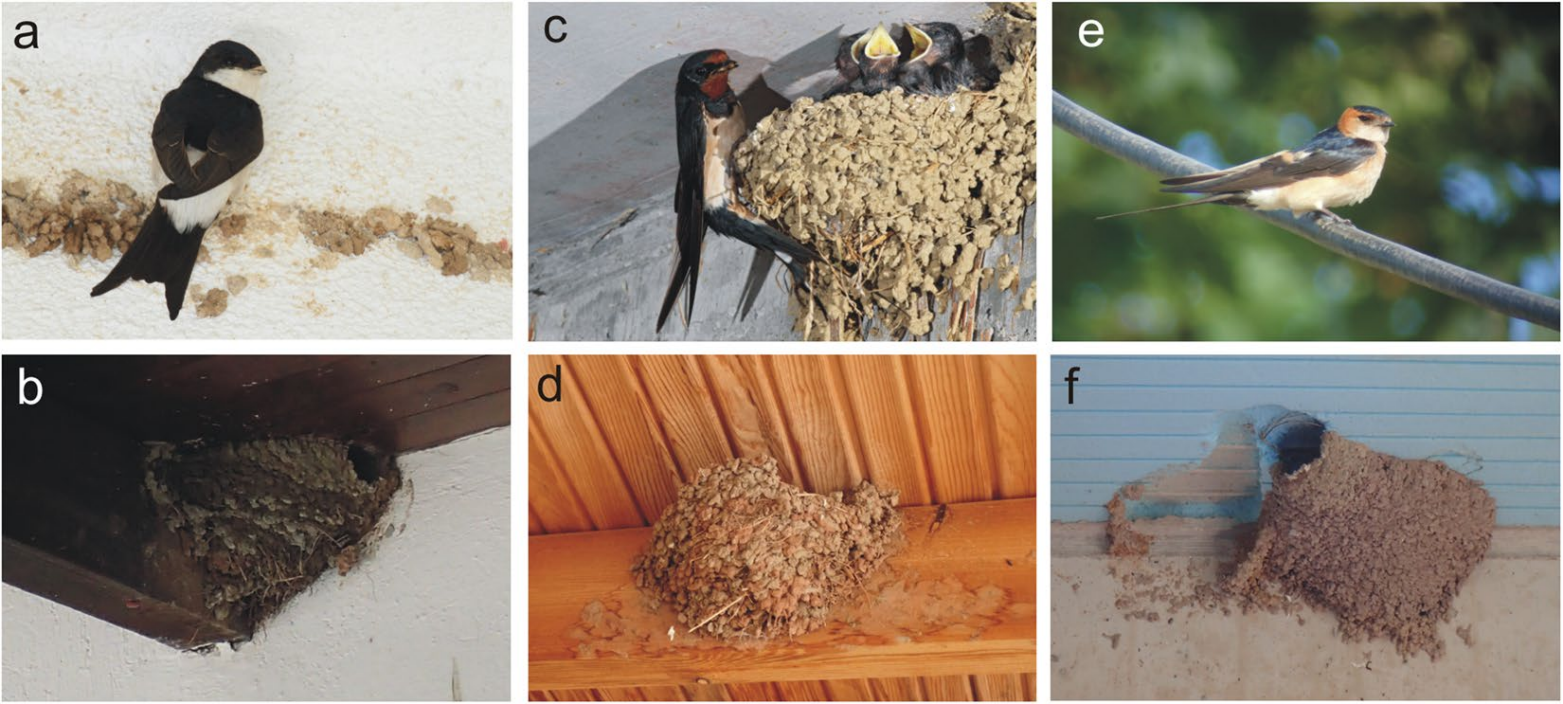
Pictures of the three different species used in this study and their nests: (**a**,**b**) the House Martin, (**c**,**d**) the Barn Swallow, and (**e**,**f**) the Red-rumped Swallow.

The last three species build mud nests in sheltered locations, principally in rural areas and human settlements, though the nest style differs among the three species^1^. House Martins are generally colonial breeders, closely associated with buildings, but large colonies can also be found on cliffs. They build an enclosed mud nest with the entrance at the top, usually attached to eaves or overhangs (Fig. 1). Red-rumped Swallows are generally solitary and breed mainly in mountainous areas with cliffs and coastal areas but also in towns and villages. They build a completely closed semi-spherical nest of mud with a tunnel entrance, under overhangs, bridges, eaves or in caves (Fig. 1). Contrary, Barn Swallows, a typical farmland species, build open-topped mud cups reinforced with straws and plant material (Fig. 1), usually adhered to beams, walls or ledges of houses, in barns or in sheds^2,3^. Mud nesting hirundines, such as the House Martins and the Red-rumped Swallows, invest enormous effort in nest construction, as they might need to carry more than 1000 pellets of wet mud within a period of a couple of weeks^4–6^.

Nest quality plays a significant role in breeding performance and success in mud-nesting hirundine species^7^, yet it has been principally studied in terms of site and habitat selection^8–11^, territory quality^12^ and the properties of nest-lining feather material^13^. However, little is known about the quality of the soil materials used for nest construction^14,15^.

Soil type plays an important role among animals that build their nests with soil materials, as they often opt for specific soil types with particular characteristics for nest construction^16^. Soil composition has been studied for burrow-nesting bird species, such as Sand Martins^16–18^, Kingfishers^19,20^ and Bee-eaters^21–23^, burrowing parrots^24^, Campo Miners^25^, mud wasps^26^, sphecid wasps^27^, burrowing spiders^28^ and termites^29,30^. It has been generally shown that burrow-nesting birds opt for sediment/soil substrates that could be easily dug and provide tunnel stability (“Heneberg Compromise”)^15^, and in particular they prefer silty sand sediments such as loess deposits^15–23^ usually enriched in Quartz^23^.

However, to date studies on soil type selection by Barn Swallows, House Martins and Red-rumped Swallows are missing, as these species do not use already existing embankments and holes but rather need to collect soil material from the adjacent region for nest construction^31^. Specific data on the texture of the building materials of hirundine mud nests have been presented before only by Kilgore and Knudsen^14^, who reported textural differences between the Cliff Swallows (*Petrochelidon pyrrhonota*) and the Barn Swallows of Western Montana. They observed that Barn Swallows preferred mud with a higher silt and lower sand content than Cliff Swallows and suggested that high sand contents improve the processing of the mud but reduce nest stability. We expect that mud-nesting hirundine species prefer fine grained sediments from loose deposits, preferably wet enough to allow them to process and make them suitable for nest building. In this paper, we attempt for the first time to investigate the soil materials used for nest construction by the sympatric species Barn Swallow, House Martin and Red-rumped Swallow in southeastern Europe, in order to identify possible patterns in the use of specific minerals and in the particle composition of the nest material. Furthermore, we aim to explore if the different species use varying soil/sediment materials depending on the architecture of their nests.

## Results

### Nest material: mineralogical composition

**Figure 2:**
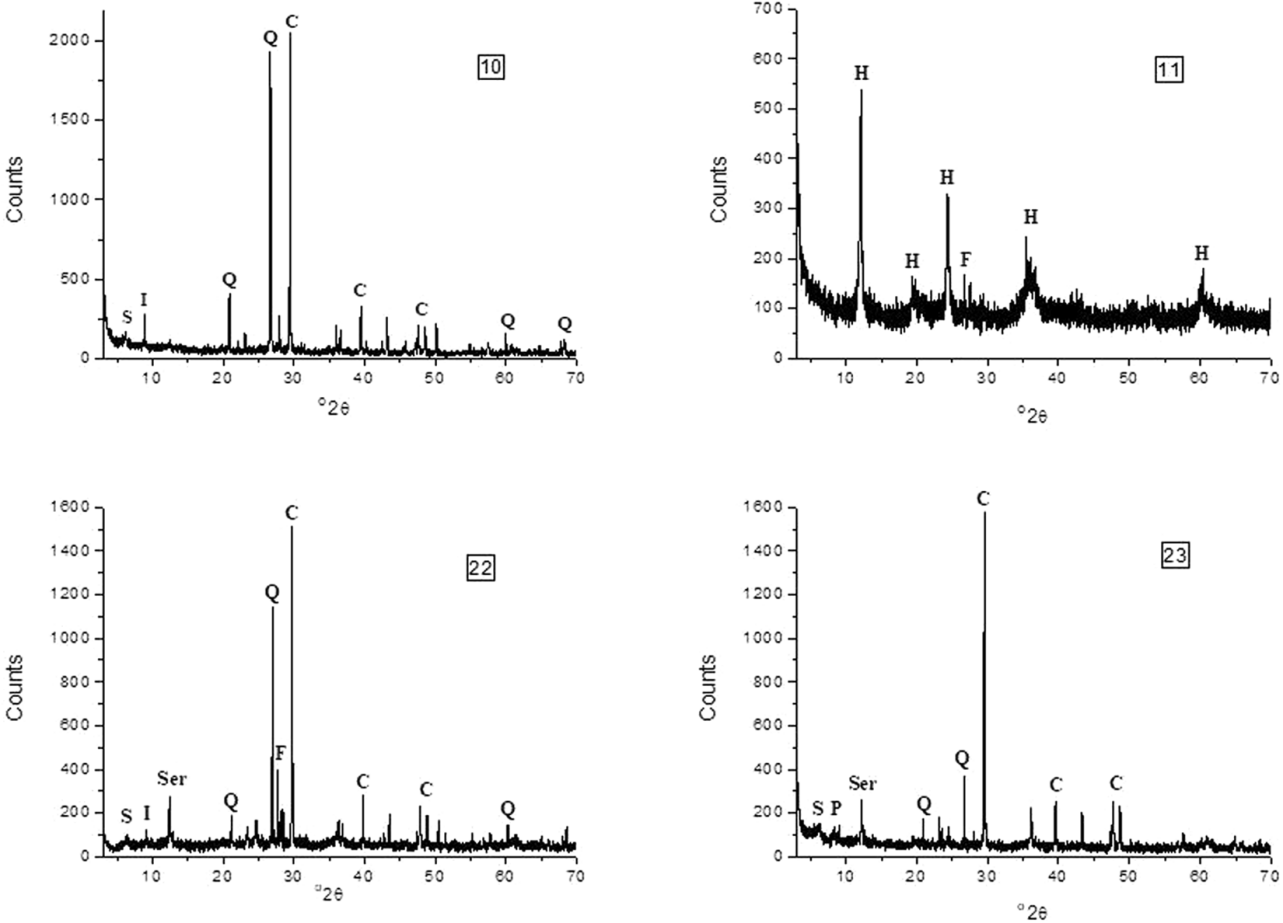
Representative X-ray diffraction (XRD) patterns showing the presence of Sm: smectite, I: illite, H: halloysite, P: palygorskite, Q: quartz, F: feldspars and C: calcite.

**Figure 3:**
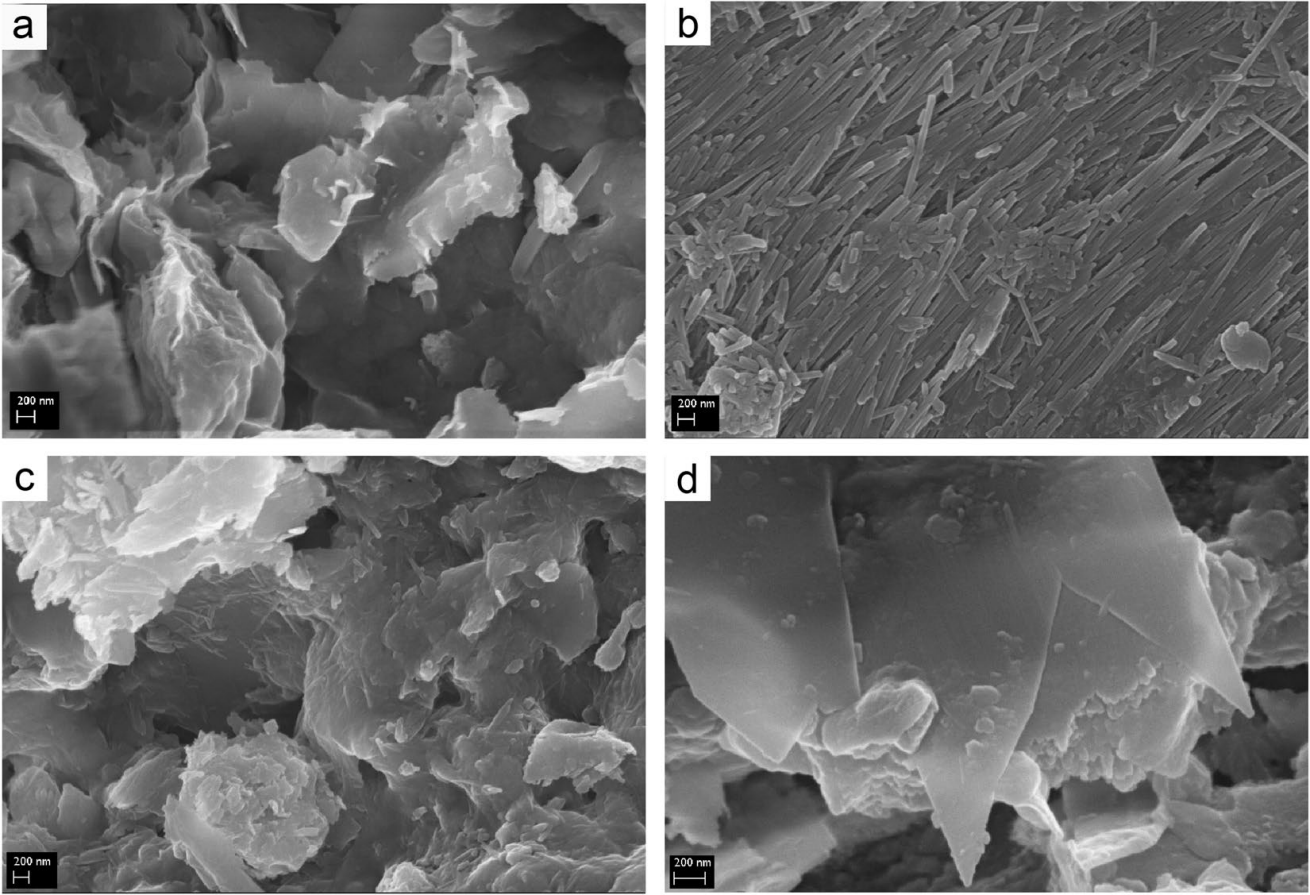
Scanning electron microscope (SEM) images of nest samples showing the presence of (**a**) smectite, (**b**) halloysite, (**c**) saponite transforming to palygorskite and (**d**) mica.

The qualitative and semi-quantitative mineralogical analyses indicated the coexistence of several clay minerals and fewer non clay minerals (Table 1). The clay minerals were in most cases smectite and illite, less commonly halloysite, kaolinite and chlorite, and occasionally palygorskite and mixed-layers. The amount of clay minerals were generally low (<22%), except for the case of halloysite observed in two nest samples (where no smectite was present; Table 1). The non clay minerals that were identified were in most cases quartz, calcite and feldspars that usually coexisted (Fig. 2). Many nests were located in areas with rocks with high amounts of other non clay minerals that are characterized by a variety of colours such as olivine (green), pyroxenes (dark colours, usually green), oxides (variety of colours) or dolomite (coexisting with calcite but usually darker than calcite). These minerals were absent from the nest samples of all studied species from all localities. Additionally, no amorphous material was detected. In a few cases only serpentine minerals (two cases) and amphiboles (one case) were detected in low amounts (9–12%). The varieties of both serpentine and amphibole minerals that were found in these nests were white or colourless (as it was indicated by macroscopic observations). Furthermore, in the nest samples of all three species no opaque minerals were detected. The study of the nest samples with a scanning electron microscope (SEM) revealed the morphology of the crystals of the minerals and the micromorphology of the nests (Fig. 3). Also, it was possible to distinguish mineral phases not able to be determined by the X-ray diffraction (XRD) analysis (below detection limits). In this way, traces of micas (Fig. 3d) as well as palygorskite fibres were detected in a sample containing smectite (saponite) from a nest in the Geohellas saponite and palygorskite factory (Fig. 2). From previous studies in these deposits^32,33^ it is known that palygorskite is formed from saponite (Fig. 3c), and thus, these minerals usually coexist in open clay mines and in the sedimentary ore piles stored in the Geohellas factory yard, one of the sampling sites, prior to processing. It was determined that halloysite (Fig. 3b) was in the form of unusually oriented long tubes (tubes placed parallel to each other) and smectite was in the form of flakes (Fig. 3a).

**Table 1:** Semi-quantitative mineralogical analyses of the nest samples (Qrtz: quartz, Cc: calcite, Feld: feldspars, Ser: serpentine, Amph: Amphibole, Sm: smectite, ill: illite, Kaol: kaolinite, Hall: halloysite, Chl: chlorite, Pal: pallygorskite) and grain size analyses (mean and sorting), as well as the respective texture. Average values of each parameter for each species are also included.

Species	Sample Code	Mineralogical Analysis (%)											Grain Size		
		Qrtz	Cc	Feld	Ser	Mica	Sm	ill	Kaol	Hall	Chl	Pal	Mean (μm)	Sorting (μm)	Texture Name
*Hirundo rustica*	1	32	0	23	0	12	16	0	0	0	15	0	5.998	3.243	MUD
	2	8	89	0	0	0	0	0	3	0	0	0	6.300	4.220	MUD
	4	33	41	0	9 (Amph)	0	11	5	0	0	0	0	26.46	6.480	sandy SILT
	10	39	39	5	0	0	5	8	0	0	4	0	38.28	6.000	sandy SILT
	12	0	0	0	0	0	2	0	0	98	0	0	63.39	6.499	sandy SILT
	14	8	50	5	0	0	15	6	0	0	0	0	32.65	5.465	sandy SILT
	15	15	33	5	0	0	22	5	20	0	0	0	35.66	8.584	sandy SILT
	16	39	6	25	0	0	9	21	0	0	0	0	8.250	4.097	MUD
	17	38	24	5	0	0	18	10	0	0	5	0	7.970	3.439	MUD
	18	68	10	15	0	0	0	8	0	0	0	0	9.563	5.395	sandy MUD
	19	30	48	6	0	0	9	8	0	0	0	0	12.07	5.353	sandy SILT
	**Average:**	**28.182**	**30.9**	**8.1**	**0.0**	**1.1**	**9.7**	**6.5**	**2.1**	**8.9**	**2.2**	**0.0**	**22.4**	**5.3**	
*Cecropis daurica*	3	58	29	5	0	0	0	8	0	0	0	0	51.49	8.705	muddy SAND
	6	53	13	0	0	0	12	7	15	0	0	0	7.396	4.565	MUD
	7	65	9	11	0	0	6	8	0	0	0	0	54.37	5.771	sandy SILT
	8	37	46	4	0	0	7	6	0	0	0	0	71.57	9.784	sandy MUD
	11	0	0	14	0	0	0	0	0	86	0	0	138.8	5.857	silty SAND
	13	61	10	17	0	3	4	5	0	0	0	0	50.31	7.863	sandy SILT
	**Average:**	**45.7**	**17.8**	**8.5**	**0.0**	**0.5**	**4.8**	**5.7**	**2.5**	**14.3**	**0.0**	**0.0**	**62.3**	**7.1**	
*Delichon urbicum*	9	52	11	9	0	0	21	7	0	0	0	0	48.29	4.606	silty SAND
	5	32	0	23	0	10	22	0	0	0	0	0	39.64	7.560	sandy SILT
	20	24	8	10	0	0	8	20	0	0	30	0	16.49	5.651	sandy SILT
	21	62	12	16	0	0	5	5	0	0	0	0	7.301	3.470	MUD
	22	26	45	7	12	0	6	5	0	0	0	0	60.17	7.991	sandy SILT
	23	10	51	0	12	0	12	7	0	0	0	8	82.96	8.900	muddy SAND
	**Average:**	**34.3**	**21.2**	**10.8**	**4.0**	**1.7**	**12.3**	**7.3**	**0.0**	**0.0**	**5.0**	**1.3**	**42.5**	**6.4**

**Figure 4:**
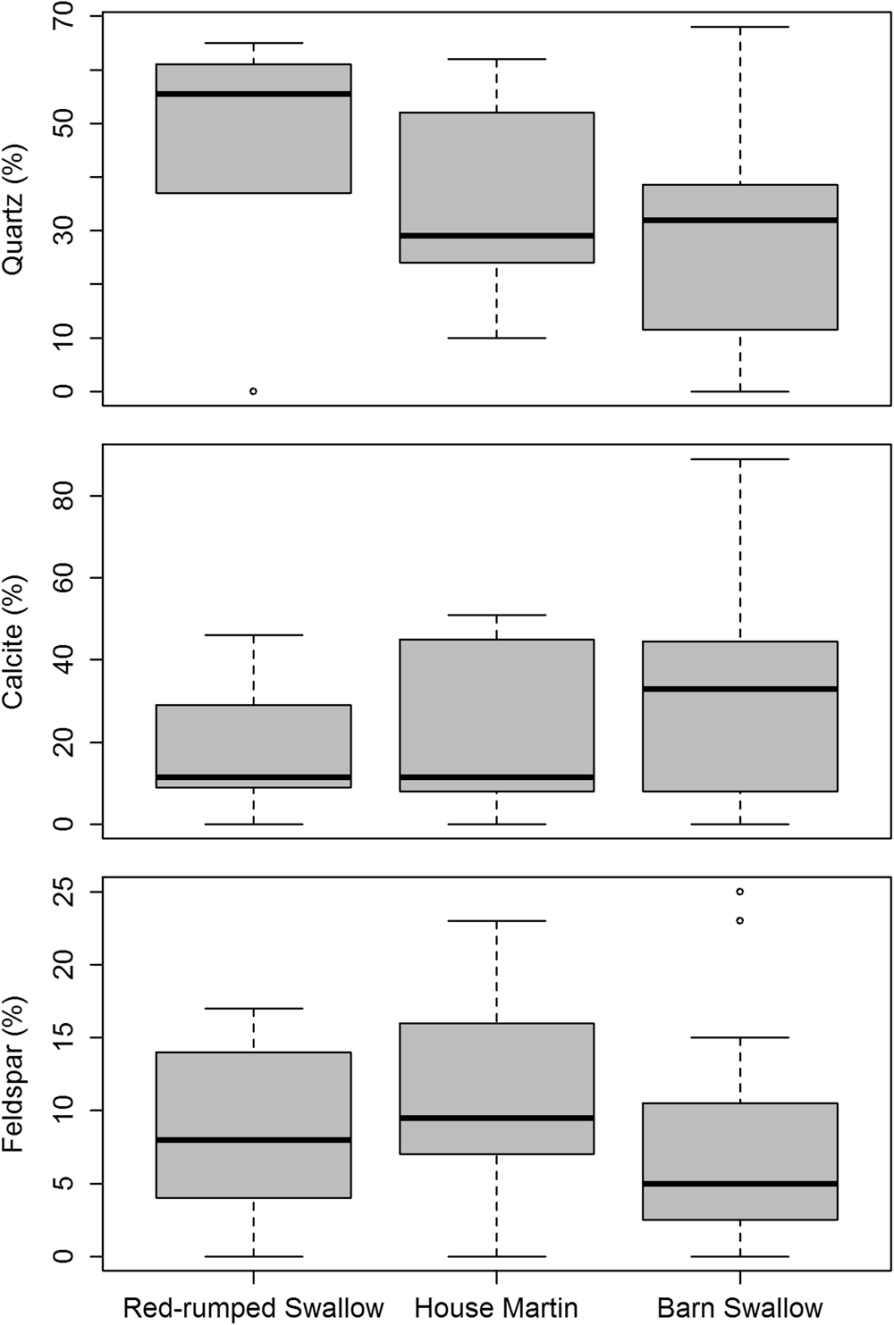
Boxplots of quartz (**a**), calcite (**b**) and feldspar (**c**) content of the nests of the three studied hirundine species.

Permutational Multivariate Analysis of Variance (PERMANOVA) did not reveal any significant differences in the total mineral composition of the nests between the three species (*F* = 0.759, *p* = 0.575). The examination of the mineralogical content of each of the three most commonly detected non-clay minerals with a Kruskal-Wallis test did not show any differences among the three species either (Qz: H = 2.36, *p* = 0.31; Cc: H = 0.58, *p* = 0.75; feld: H = 0.99, *p* = 0.61; Fig. 4a–c); hence, no species driven trend in mineral use was observed.

### Nest material: grain size

**Figure 5:**
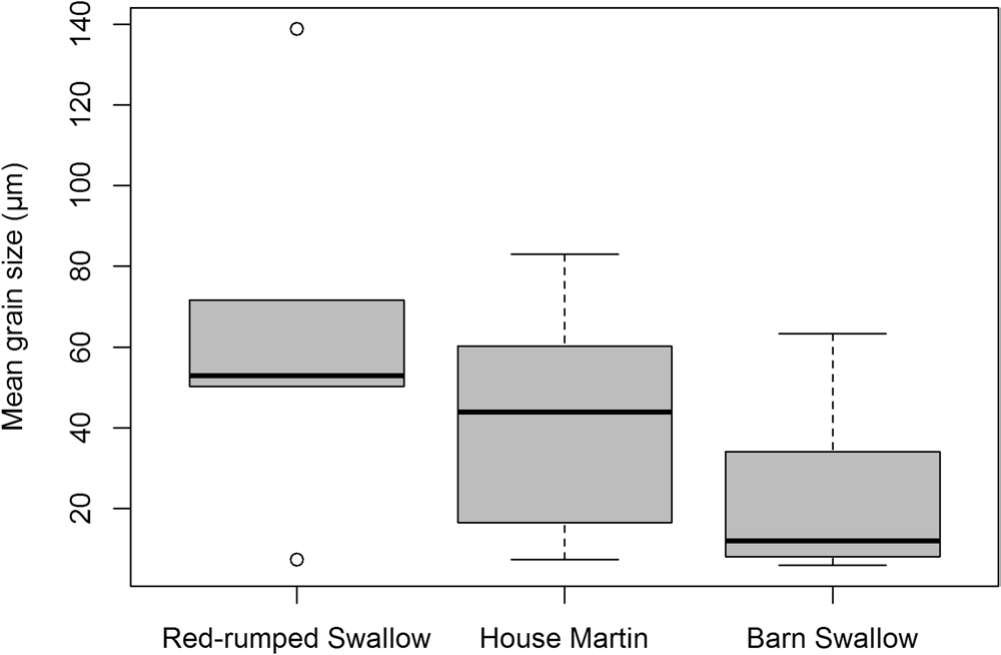
Boxplots of the mean grain size of the nests of the three studied hirundine species.

Grain size analyses for each species’ nests (mean grain size and sorting (-see Material and Methods section)) indicated that the main textural characteristic of the nests was sandy silt and mud (Table 1). The mean grain size of the nests ranged between 5.9 and 138 μm with an average value of 36 μm. The coarsest material was used by the Red-rumped Swallow (average value of the mean nest grain size ± standard error: 62.3 ± 17.6 μm), the finest material was used by the Barn Swallow (22.4 ± 5.6 μm), while the mean grain size was intermediate for the House Martin (42.5 ± 11.4 μm; Table 1, Fig. 5). Nevertheless, the Kruskal-Wallis test indicated that there was no significant difference in the mean grain size among the three species (H = 5.7, *p* = 0.057). Pairwise comparisons between the species showed that there was statistically significant difference between the Barn and the Red-rumped Swallow (Mann-Whitney U = 12, *p* = 0.039), whereas no significant difference was found between the Red-rumped Swallow and the House Martin (Mann-Whitney U = 12, *p* = 0.378) and between the Barn Swallow and the House Martin (Mann-Whitney U = 17, *p* = 0.193).

**Figure 6:**
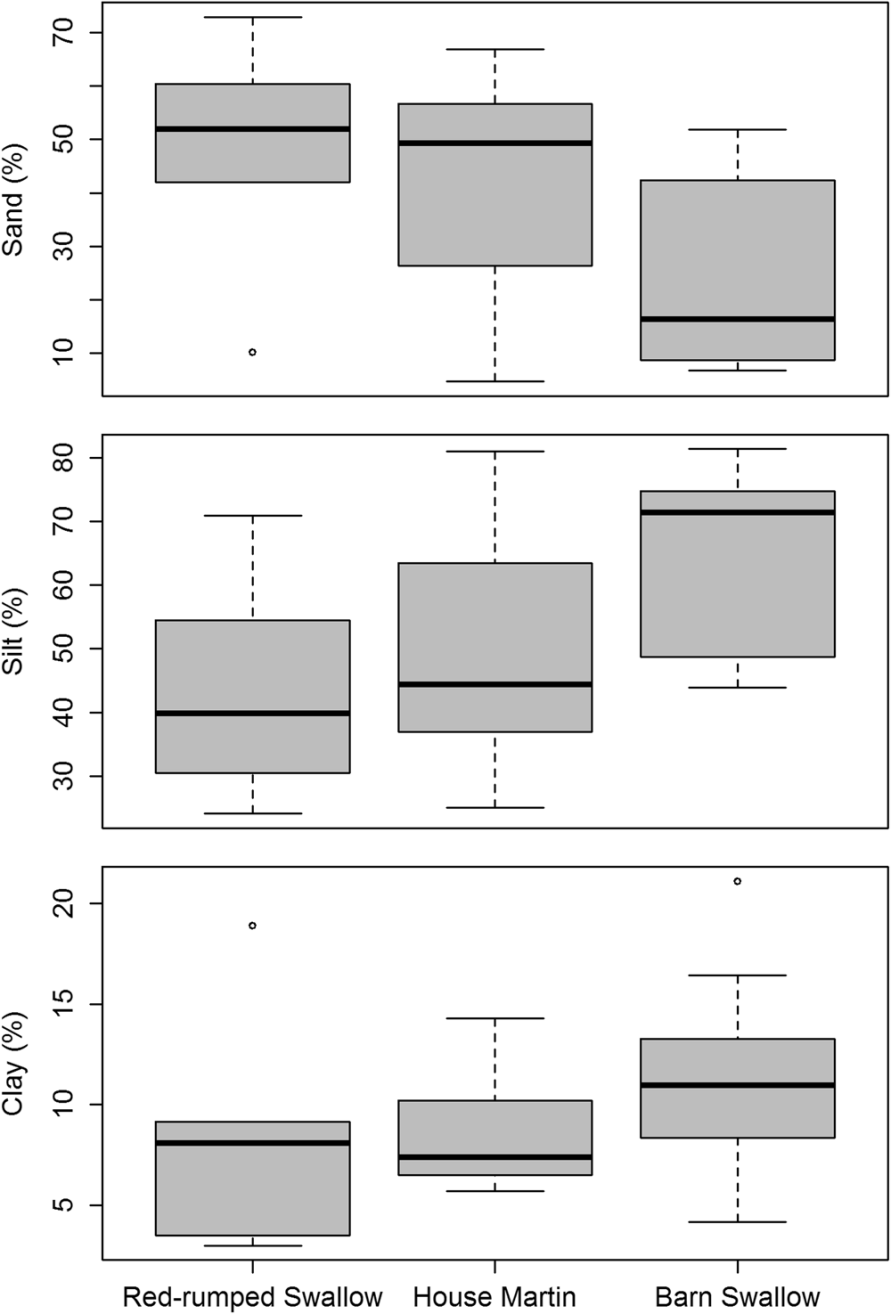
Boxplots of the Sand (**a**), Silt (**b**) and Clay (**c**) percentages of the nests of the three studied hirundine species.

It seems that all species mainly used clay and silt with a grain size <63 μm. In the nest samples of all species the portion of very fine to medium sand (62.5–500 μm) percentage was always below 50% (4.2–48.1%), and the coarse to very coarse sand percentage (500–2000 μm) was usually low or extremely low (0–24.7% but mostly below 10%) (Fig. 6a). The silt percentage usually dominated ranging from 24.2–81.3% (Fig. 6b), whereas the clay percentage was always rather low (3–21.1%) usually with values below 13% (Fig. 6c). From the three box plots (Fig. 6) it is clear that Barn Swallow nests are characterised by fine grain sizes and particularly by silt, Red-rumped Swallow nests by coarser grain sizes, whereas Barn Swallow nests by intermediate values. Again though, a Kruskal-Wallis test indicated that there was no significant difference in any of the grain percentages among the three species. However, pairwise comparisons between the species showed that there was statistically significant difference between the Barn and the Red-rumped Swallow for the sand and silt percentages (Mann-Whitney U = 11, *p* = 0.031 and U = 9, *p* = 0.018 respectively) but not for the clay percentage. The best sorting of the grains (as indicated by the lowest average of the grain size standard deviation values) was observed in the nest samples of the Barn Swallow (5.3 μm), whereas the poorest sorted material was observed in nests of the Red-rumped Swallow (7.1 μm), while those of the House Martin presented an intermediate value (6.4 μm). Nevertheless, the observed difference in the sorting of the grains was not statistically significant (Kruskal-Wallis H = 3.32, *p* = 0.19).

### Relationship between mineral content and grain size

**Figure 7:**
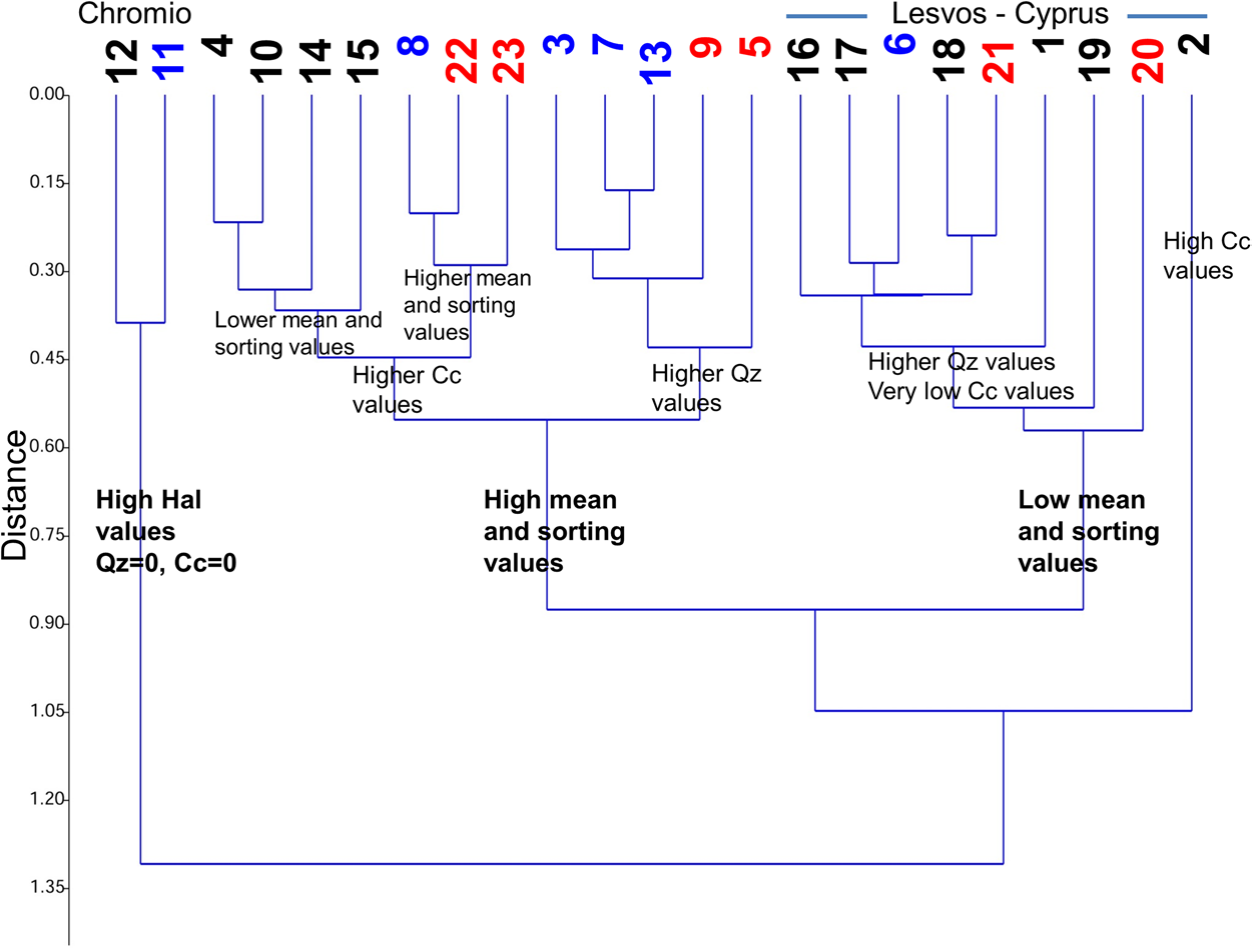
Cluster analysis plot. Black nest samples: Barn Swallow, red nest samples: House Martin, blue nest samples: Red-rumped Swallow.

**Figure 8:**
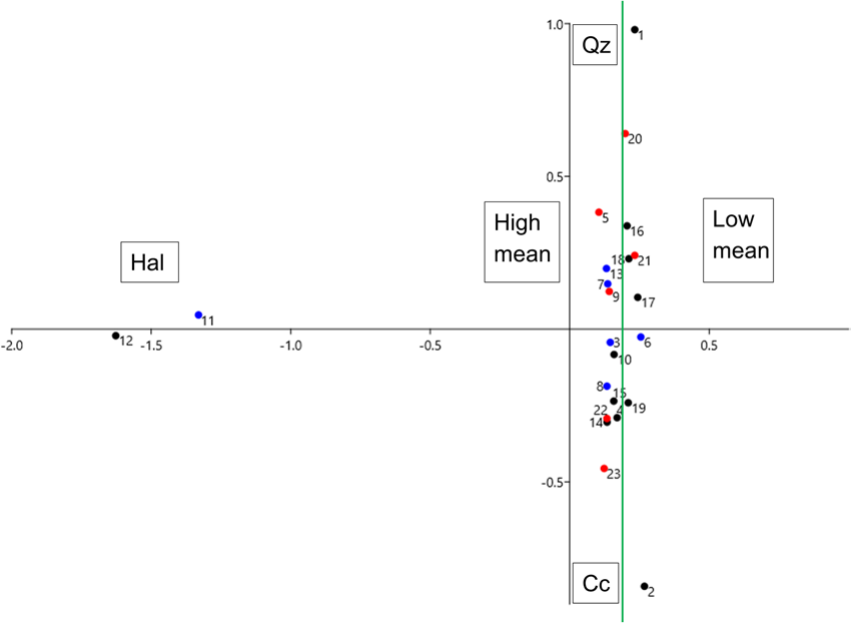
Correspondence analysis scatter plot. Black dots: Barn Swallow, red circles: House Martin, blue x: Red-rumped Swallow. The Hal rich nest samples are clustering separately at the far left side of the plot. On the left of the green line nest samples with high mean grain size values are found, whereas on the right those with low mean values are found. Towards the upper part of the plot nest samples with increasing contents of Qz are represented, whereas towards the lower part of the plot nest samples with increasing Cc contents are found respectively.

The cluster analysis showed that the studied nest samples were segregated into three main clusters (Fig. 7), which correlate well with the distribution of the nest samples in the correspondence analysis plot (Fig. 8) along the X axis (high Hal content values, high mean and low mean values). Cluster I was characterized by high Hal content values and zero Cc and Qz, while the other two clusters were divided depending mostly on grain size and sorting values. Clusters II and III can be further subdivided into subclusters mainly because of the differences in Cc and Qz values (Fig. 7). None of the main clusters contained nests from only one species distinguishing its nest mineral composition from the other two.

Cluster II can be further divided into two sub-clusters (A and B) on the basis of the higher Cc and Qz contents respectively, indicating generally that Barn Swallow nests show a preference to higher Cc contents and Red-rumped Swallow nests to higher Qz. In cluster III the main subcluster is characterised by higher Qz and relatively low Cc values.

Correspondence analysis (axis 1: 41.5%, axis 2: 19.5%) seems to verify in general the cluster analysis groupings (Fig. 8). The high Hal nest samples grouped together to the left of the scatter plot are well separated from the main cluster. In the main group of nests, samples with high mean and sorting values are located to the left side while those with low mean and sorting values are on the right. Also, nest samples with high Qz and Feldspar contents are found to the upper right quadrant, whereas those with high Cc contents are placed in the lower right quadrant.

As the effect of high Hal nest samples (apparent outliers in the cluster analysis and CA) could be masking the possible differences among other nest samples, we performed additional statistical comparisons of the mineralogical and sedimentological characteristics excluding the Hal enriched nest samples from Chromio. In this case, the mean grain size presented a marginal trend towards a significant difference among the three species (Kruskal-Wallis H = 6.23, *p* = 0.044), whereas the observed differences in the sorting of the grains and the Qz content were not statistically significant (Kruskal-Wallis H = 3.84, *p* = 0.146 and H = 4.97, *p* = 0.083, respectively). Pairwise comparisons performed with Mann-Whitney U-test between the species showed that there was a marginally significant difference in the mean grain size between the Barn and the Red-rumped Swallows (U = 8, *p* = 0.043) but a non-significant trend between the Barn Swallows and the House Martins (U = 12, *p* = 0.058). Using these nest samples (excluding the high Hal ones) similar marginally significant difference was found in the Qz content between Barn and Red-rumped Swallows (U = 8, *p* = 0.043), but a non-significant difference was observed between the Red-rumped Swallow and the House Martin (U = 5, *p* = 0.083).

## Discussion

Although the soil composition of burrow-nesting bird species, such as Sand Martins^16–18^, Kingfishers^19,20^ and Bee-eaters^21–23^ have been already studied, it seems that until now almost nothing similar had been done for bird species building mud-nests such as Barn Swallows, House Martins and Red-rumped Swallows, with the exception of Kilgore and Knudsen^14^ work on the Cliff Swallows and Barn Swallows of Western Montana. Furthermore, nest studies from a sedimentological and stratigraphical perspective are very scarce^14,15,23^. Therefore, herein we report for the first time mineralogical and sedimentological data from nests of these three common and sympatric mud-nesting hirundine species from several localities of southeastern Europe, indicating that the three species present different nest building strategies and depending on the nest architecture, each of them seems to show preference towards specific minerals, based on their properties and specific grain size.

The qualitative as well as the semi-quantitative mineralogical analyses of the nest samples of all species from all locations show some notable similarities. The minerals used by all three species in terms of building materials can be divided into two categories: a) minerals used as aggregates are materials with low plasticity indices and b) minerals used as cement or as glue are materials with high plasticity indices. The plasticity index (P.I.) measures the plasticity of a soil and more specifically the range of moisture in which the soil presents plastic properties. Therefore, minerals with high plasticity indices used as cement provide more strength and stiffness to the nest building materials, allowing the birds to build structurally sound mud nests^23^. Our results show that from the minerals that were identified in the nest samples only clay minerals can be used as cement due to their specific plasticity properties and especially their high plasticity index. Besides, proper cementation is a key-factor for securing nest particle cohesion^15^ and clay is considered to be a suitable material for this purpose^14,23^. It was determined that only specific clay minerals were used as cement, whereas others were not preferred even if they were accessible. More specifically, all species use active clay minerals of the smectite and illite groups as cement (Figs 2 and 3). If these minerals are not present or easily accessible in the broader area, they alternatively use halloysite, kaolinite or chlorite (Fig. 2), as in two cases (Chromio) where halloysite was used instead as cement (Figs 2(c,d) and 3b). In these cases, the amount of halloysite is very high, as it was the only mineral identified by X-ray diffraction (XRD) or coexists with low amounts of smectite. Halloysite is used both as cement and as aggregates as well. It is evident that the amounts of clay minerals are generally low, in most cases less than 30% and generally in the range of around 15%, which is in accordance with the findings of Kilgore and Knudsen^14^, indicating that the studied species can accurately identify the properties of the minerals. The mineralogy of the nest reveals the plasticity of the cement materials indirectly. It is known that the plasticity index (and liquid limit) of smectite (a mineral with high plasticity index) is up to about 7–15 times higher than halloysite’s and kaolinite’s indices^34,35^. Low clay mineral contents suggest significant contribution of smectite in the clay fraction of the nest materials. In the cases of smectite absence (or its presence in very low amounts), the amounts of halloysite and kaolinite determined in the nests are in agreement with this difference in mineral plasticity: the amount of halloysite is about 90%, i.e. about 5–15 times higher than the amount of smectite in almost every case. An exception to the above statement is nest sample 2, where smectite is absent and the amount of kaolinite is very low. In this case, the dominant microcrystalic calcite seems to be sufficient for nest stability and thus, no cement material is used.

In most cases more than one clay mineral are present in the nests. This is probably not interpreted as a choice of the species rather than the usual coexistence of different clay minerals in soils, rocks, clay occurrences and even clay mineral deposits. Purification of a clay mineral from a clay sample is a very difficult procedure and particularly, in cases where it coexists with other clay minerals^36^. Therefore, the coexistence of different clay minerals is a result of the distribution of the respective clay minerals generally in nature and in the broader areas of the nests. Another interesting observation is the preference for saponite (a member of the smectite group which thus possesses a higher plasticity index), rather than palygorskite in the area where they coexist (palygorskite and saponite deposits of Grevena). In these cases, palygorskite is found in very low amounts and is probably due to the general preference of all three species to smectite minerals (including saponite) over all the other clay minerals.

The most common and easily accessible non clay minerals were quartz, calcite and feldspars (Fig. 2). Our results indicate that the studied species preferred such colourless and white minerals avoiding opaque minerals. Despite the fact that some nests were in areas where rocks with high amounts of other minerals of varying colours such as olivine, pyroxenes, oxides or dolomite are available, all three species seem to ignore them and instead use colourless or white minerals. In particular, quartz and calcite were found in varying percentages in all studied nest samples except for the Halloysite rich nest samples from Chromio. Quartz is considered an important mineral for nest cohesion, used also in other hirundine species^15^, and in high quantities by wasps as well^26^. The three species included in this study not only seem to prefer these two minerals, but some trends can be also identified particularly for the quartz contents; the Red-rumped Swallow presents the highest average quartz values (45.7%) followed by the House Martin (34.3%), whereas the Barn Swallow has the lowest values (28.2%). Excluding the Halloysite rich nest samples from the statistical analyses, a significant difference in quartz use is noted only between the Barn and the Red-rumped Swallow. Therefore, the Red-rumped Swallow shows a preference to quartz, which could be also considered as a biased result due to the small sample size and the local rock formations that provide the respective minerals in the sampled localities. A similar but statistically not significant trend can be observed for the calcite. The Barn Swallow presented the highest average values (30.9%) followed by the House Martin (21.2%), whereas the Red-rumped Swallow had the lowest values (17.8%). The observed trends for the two minerals can be also observed in the cluster and correspondence analysis plots (Figs 7 and 8), where the segregation of clusters II and III in sub-clusters is mainly affected by the quartz and calcite contents of the nest samples. Thus, not only is there a preference for colourless and white minerals, but there is also a preference for quartz and calcite in particular (since these minerals are present in almost all the nest samples and in high percentages in most of the nest samples, Table 1).

It should be also noted that no oxides were detected, even though oxides are common materials in soils (especially iron oxides) as well as around the sampling sites. Additionally, no amorphous material was detected, despite the fact that it was often available in the broader area of the nests. The preference for white and colourless minerals could be attributed to the fact that coloured and especially dark coloured minerals absorb light and thus, get more easily heated. For the same reason the studied species seem to avoid opaque minerals, even though this could also be a result of the fact that all opaque minerals are rare in the studied areas, except for the presence of chromite in Grevena area.

Another significant observation is that the main minerals used by all three species have low specific gravity values, while minerals with high values are absent. More specifically, quartz, feldspars, calcite as well as clay minerals have specific gravity values lower than 2.8. The common minerals that all species avoid have specific gravity values higher than 2.8 such as the dolomite (approx. 2.85), the amphiboles and pyroxenes (>3 depending on the mineral), the olivine (about 3.3), while opaque minerals have even higher values (depending on the mineral; e.g. chromite has about 4.6). The low specific gravity could be a significant factor for the stability of the nests since heavy nests would be more liable to collapse^15^.

Grain size distribution analyses revealed that the amount of the clay grain size percentage is relatively low in the nest samples of all studied species (Fig. 3). The main textural characteristics of the nests were sandy silt and mud. This finding is in relative congruence with the findings of Kilgore and Knudsen^14^, who also showed that Cliff and Barn swallows had a preference for sandy and silty materials with low clay sized components, as it is the case with the soil texture of the preferred nesting substrates of burrowing birds^15–23^. Additionally, the amount of larger grain size particles decreases, when the size of the non clay minerals is smaller rather than depending on the amount of clay minerals (which was generally low in most nests). Also, there was a statistically significant difference among the mean grain size and the sand and silt contents of the nests which was particularly evident between the Barn and the Red-rumped Swallow, but weak between the Barn Swallow and the House Martin, indicating that the three species use different grain sizes. Particularly the Red-rumped Swallow (except sample 6) preferred coarser grain sizes, while the Barn Swallow showed preference for finer grain sizes (average mean grain size 22.4 μm). The later was also observed by Kilgore & Knudsen^14^ for Barn Swallows. As mentioned above, the Barn Swallow uses organic material to build and reinforce its open-topped cup nests. Hence, it seems reasonable to use finer grained material as cement to connect them, as the former will possibly increase the structural strength of the nest^37^. On the other hand, the closed semi-spherical nests of Red-rumped Swallows and House Martins are built only with mud, and therefore, coarser grains (mean average 62.3 and 42.5 μm respectively) of colourless and white minerals are possibly used as aggregates to reinforce the sturdiness of the nests. According to Kilgore & Knudsen^14^ such coarser aggregate mixtures used by Cliff Swallows to improve the workability of the mud, facilitating the building of complex shaped nests, which seems to be the case for the nests of Red-rumped Swallows and House Martins.

Again, there are certain trends that can be identified in the sediment properties of the studied nests linked with certain species, influenced by the grain size and the mineral properties of the nest building materials. This is also supported by the fact that birds often choose to travel rather long distances in order to collect the suitable materials ignoring other surrounding sources^6,38^. However, the local rock formations, which provide the respective sediments in each locality through weathering processes also play a major role in the mineral and grain size content of the nests^14^, as it is depicted in the cluster and correspondence analysis plots (Figs 7 and 8), where the segregation into clusters II and III is mainly affected by the grain size of the nest samples (cluster II: high mean grain size and sorting; cluster III: low mean grain size and sorting). Notwithstanding the fact local geology could affect the grain size of the available nest material in each sampled locality, it is evident that the Barn Swallows are consistently selecting smaller grains, while Red-rumped Swallows larger. Among the samples there are three cases of nest samples from sympatric species found next to each other or in close proximity: 6 nest samples from Damandri monastery (4 Barn Swallow and 2 House Martin nests found next to each other), 2 nest samples from Chromio (where the Barn Swallow nest was found less than 100 m away from the Red-ramped Swallow nest) and 3 nests from the wider area of Rio (one nest sample from each species), where the distances are greater (the distances between the nest sites range from 1.7 to 5.5 km) but the lithology of the surrounding rocks is the same. In all three cases we got consistently different mean grain sizes following the same pattern: Barn Swallows presented the lowest and Red-rumped Swallows the highest mean grain size. Characteristically in the case of Chromio, the only site where the nests from the two sampled sympatric taxa contained Hal in extremely high proportions (86% and 98% respectively), the mean grain size was notably high for both nest samples but again the Barn Swallow nest sample provided a mean grain size of 63.39 μm (the highest by far among the Barn Swallow nest samples), which was though significantly lower than the Red-rumped Swallow value of 138.8 μm.

Similarly, burrowing nest building birds possibly have the ability to select suitable soil substrates for nest building not only based on their physical properties^15,16,21,23^ but also on their grain size^18–20^ and possibly on their mineral properties. It has been shown that sandy to silty substrates such as loess are preferable substrates for Bee-eaters^23^ and Sand Martins^15,18^, and that even slight differences in the clay percentage affect the selection of nest substrates by Malachite Kingfishers^20^. In the former case loess substrates consist mainly of silt sized quartz particles^23^, therefore it may be possible that except for mud nesting birds, burrowing birds could somehow recognize the properties of quartz and clay minerals among others.

## Conclusions

The three studied species, the House Martin, the Barn Swallow and the Red-rumped Swallow use clay minerals as cement for nest building and preferably smectite and illite. If they are absent they alternatively use halloysite, kaolinite or chlorite. The amount of clay minerals are generally low increasing when clay minerals with low plasticity are available, indicating that the studied species can accurately identify the properties of the used minerals. The used non clay minerals are the common, easily accessible colourless or white ones with specific gravity values <2.8. Grain size distribution analyses revealed that the amount of clay grain size is relatively low in all species, while the amount of larger grain size particles decreases when the size of the non-clay minerals is small. To a certain extent, despite the effect of local geology the three different species seem to have certain preferences. The Red-rumped Swallow shows an increased preference for larger grain size particles and quartz, whereas the Barn Swallow favours finer grain size particles and calcite, and the preferences of the House Martin are in between the other two species.

## Material and Methods

### Study area and nest sampling

**Figure 9:**
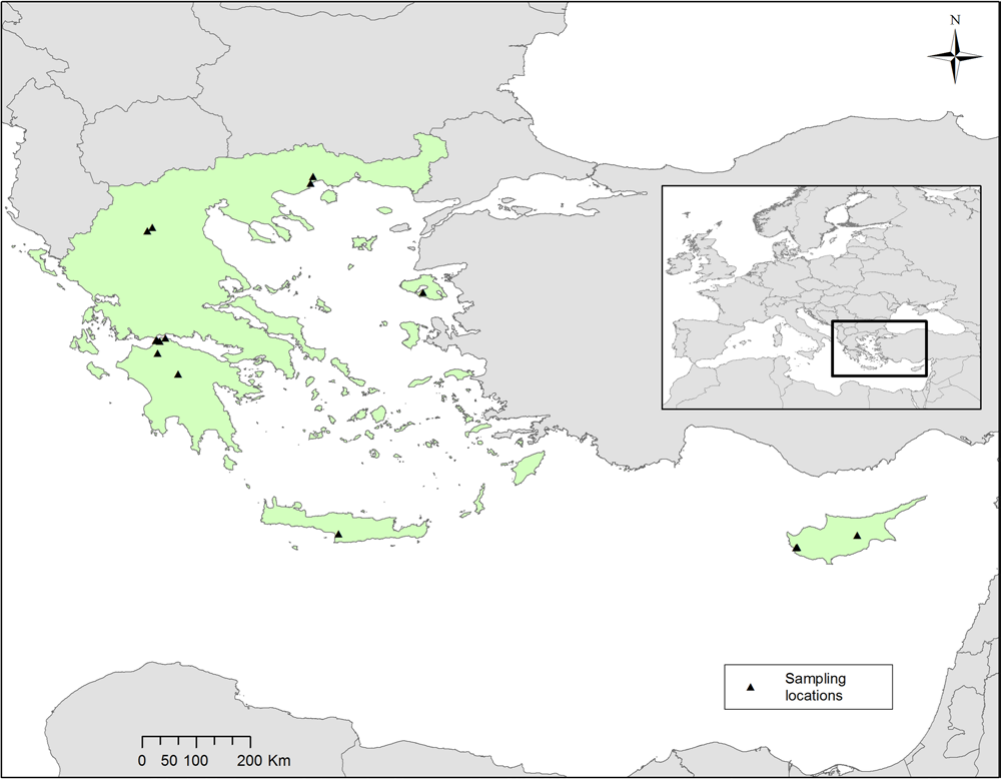
Hirundine nests were sampled from 15 localities in Greece and Cyprus.

Hirundine nest samples were collected from 23 old, abandoned or semi-destroyed nests from 15 localities distributed across Greece and Cyprus (Table 2) during 2014–2015 (Fig. 9). 11 nest samples were taken from Barn Swallow nests, 6 from Red-rumped Swallow nests and 6 from House Martin nests respectively. The soil/sediment materials from the 15 localities originated from a variety of rock formations and mainly consisted of weathering products. The source rocks in the adjacent areas can be separated into two main groups, sedimentary and igneous rocks. Most sedimentary rocks consist of marls, clays and generally of alluvial deposits of Neogene and Quaternary age (Tala, Potamia, St Helen Cave, Valmantoura Cave, Panagopoula, University of Patras, Kastelokampos, Ano Kastritsi, Voroi; Table 2), whereas in Mpravovikos Spring the source rocks are Cretaceous and Paleogene limestone and flysch deposits. The weathered igneous rocks provide fine grained sediments in the remaining localities. More precisely, at Palio the surrounding bedrocks are granites, at Damandri Monastery ignibrites, while at Chromio and Geohellas plant they are ophiolites. Furthermore, at Geohellas plant palygorskite and saponite material is stored for industrial processing. Also, Tala is located relatively close to Mount Troodos which is well known for its ophiolites. Therefore, as the sampled nests come from different localities, hirundine individuals were expected to construct them with soil/sediment materials collected from a variety of sediments. The sampled material was chosen from the upper external rim of the nests in order to avoid potential contaminants from faeces and other material deposited during the nesting period. It should be noted that the time the mineral material was exposed in the nests is too short for significant weathering effects to occur and to observe any alteration of the respective minerals, taking into consideration that the used materials are soils already exposed for a long time to weathering.

**Table 2:** Nest samples and localities of hirundine nests sampled in southeastern Europe in 2014–15.

Species	Sample code	Latitude	Longitude	Locality	Country
*Hirundo rustica*	1	34.846290°	32.446182°	St Neophytos monastery, Tala, Pafos	CY
*Hirundo rustica*	2	34.840229°	32.430886°	Tala, Pafos	CY
*Hirundo rustica*	4	35.044141°	33.446281°	Potamia, Nicosia	CY
*Hirundo rustica*	10	38.286323°	21.771007°	Kastelokampos, Achaia	GR
*Hirundo rustica*	12	40.168380°	21.713551°	Chromio, Grevena	GR
*Hirundo rustica*	14	35.068441°	24.811267°	Voroi, Crete	GR
*Hirundo rustica*	15	35.068441°	24.811267°	Voroi, Crete	GR
*Hirundo rustica*	16	39.084296°	26.221762°	Damandri monastery, Polychnitos, Lesvos	GR
*Hirundo rustica*	17	39.084296°	26.221762°	Damandri monastery, Polychnitos, Lesvos	GR
*Hirundo rustica*	18	39.084296°	26.221762°	Damandri monastery, Polychnitos, Lesvos	GR
*Hirundo rustica*	19	39.084296°	26.221762°	Damandri monastery, Polychnitos, Lesvos	GR
*Cecropis daurica*	3	38.075980°	21.798876°	Valmantoura Cave, Achaia	GR
*Cecropis daurica*	6	37.729499°	22.142662°	Mpravovikos Spring, Agridaki, Arkadia	GR
*Cecropis daurica*	7	41.014969°	24.387302°	St Helen Cave, Zygos, Kavala	GR
*Cecropis daurica*	8	38.326122°	21.930111°	Panagopoula, Achaia	GR
*Cecropis daurica*	11	40.168380°	21.713551°	Chromio, Grevena	GR
*Cecropis daurica*	13	38.272405°	21.834407°	Ano Kastritsi, Achaia	GR
*Delichon urbicum*	5	40.905568°	24.348849°	Palio, Kavala	GR
*Delichon urbicum*	9	38.292086°	21.789628°	University of Patras, Rio, Achaia	GR
*Delichon urbicum*	20	39.084296°	26.221762°	Damandri monastery, Polychnitos, Lesvos	GR
*Delichon urbicum*	21	39.084296°	26.221762°	Damandri monastery, Polychnitos, Lesvos	GR
*Delichon urbicum*	22	40.107426°	21.629467°	Geohellas plant, Grevena	GR
*Delichon urbicum*	23	40.107426°	21.629467°	Geohellas plant, Grevena	GR

### Ethics statement

Our sampling protocol was designed with respect to the species’ biology and ecology, setting as a priority not to cause any disturbance to the birds’ breeding performance. All studied populations are long-distance migrants overwintering in sub-Saharan Africa, thus, we sampled nests during autumn and winter in order to avoid potential disturbance. As Barn Swallows and House Martins have been showing declining population trends during the last decades in Europe^39^, we avoided destroying any intact nests, as it is a common practice for the three studied species to re-use the same nests year after year. Therefore, nest samples were principally taken from old, abandoned or partly destroyed nests (15 nests), which apparently had not been used for a number of successive years, to avoid damaging recently used nests. On a complementary basis a number of other nests (8 nest samples) seemingly disused, but not in a condition so as to certainly preclude future use, were sampled. For this, the minimum amount of mud material required for the mineralogical analysis (20 g) was taken and it was removed from the nest rims to allow potential repairs during future breeding seasons. Nest sampling was conducted in accordance to Greek legislation (Presidential Decree Π.Δ 0.67/81, article 6, par. 2; Ministry of Environment and Energy – document number 170670/935).

### Methods

The mineralogical analyses of the nest samples were conducted using X-ray diffraction (XRD, Bruker D8 Advance) with Cu-Kα radiation (λ = 1,54 Å) and a Nickel filter was used for the characterization of the nest samples. Qualitative mineralogical analyses were performed with the DIFFRACplus EVA12® software (Bruker-AXS) based on the ICDD Powder Diffraction File of PDF-2 2006, while semi-quantitative analyses were performed using the same software, based on the calculation of the Area method. This method is used to quantify the mineral content from diffraction pattern of an XRD analysis by measuring the diagnostic peak areas of each mineral, considering the full width at half maximum (FWHM) and corrected it by empirical factors according to Galán and Martín Vivaldi^40^, Martín-Pozas *et al*.^41^ and Schultz^42^. The detection limit of XRD is about 2–3%, thus mineral phases less than 2% cannot be detected, and therefore the analyses can be considered as semi-quantitative rather than quantitative. The morphology of the mineral grains was studied using a JEOL JEM-2100 Scanning Electron Microscope (SEM) at an acceleration voltage of 200 kV. Carbon coated grids with 200mesh were used for this method. In order to define the type of the geo-material (soil/sediments) that hirundines used for their nest construction, grain size analysis in all the collected nest samples was performed. Soil/sediments grain size classification and grain size distribution were made using a Malvern Mastersizer 2000 laser diffraction particle size analyzer and percentages of sand/silt/clay^43^ 62.5–2000 μm, 4–62.5 μm and <4 μm respectively were obtained. The USDA soil and sediments classification ternary diagrams were used to define the texture name of the geo-material that the swallows used. Based on Folk and Ward^40^ and using the GRADISTAT V.4 software^44^, the following statistical parameters used in grain size analysis were calculated for the nest materials, mean grain size (M_G_-μm) and sorting (σ_G_-μm).

### Data analysis

As one of the main assumptions of parametric tests, that of normality of distribution, was not satisfied for all studied mineralogical and sedimentological parameters, and due to the relatively small sample sizes involved in this study as well, non-parametric statistical analyses were used.

To test for possible differences in nest mineral composition among the three hirundine species, Permutational Multivariate Analysis of Variance (PERMANOVA^45^) based on Euclidean distances was performed, using the function ‘adonis’ of the ‘vegan’ package^46^ in R 3.3.2. A permutation test with 999 permutations was applied to test the null hypothesis -i.e. that no differences occur among the three species^45^.

Subsequently, we investigated potential differences of the most important minerals (those with the higher percentages in the nest samples) and soil/sediment preferences among the three species. For this, comparisons of the abundance of the three most commonly used minerals (quartz, calcite and feldspar), of the mean grain size and of the sand, silt and clay percentages were performed using Kruskal-Wallis (to compare all three hirundines) and Mann-Whitney tests (for pairwise comparisons).

With the aim to detect relations and identify groups of hirundine nests with similar mineralogical and sedimentological characteristics, a cluster analysis of these data based on the Euclidean similarity index was performed. Next, we attempted to detect emerging patterns and identify factors shaping nest positioning and groupings with a Correspondence Analysis (CA). Both clustering and CA were carried out using the software PAST 3^47^.
